# Green Tea Waste as an Efficient Adsorbent for Methylene Blue: Structuring of a Novel Adsorbent Using Full Factorial Design

**DOI:** 10.3390/molecules26206138

**Published:** 2021-10-11

**Authors:** Marwa El-Azazy, Ahmed S. El-Shafie, Bayan Al-Shaikh Yousef

**Affiliations:** Department of Chemistry and Earth Sciences, College of Arts and Sciences, Qatar University, Doha 2713, Qatar; aelshafie@qu.edu.qa (A.S.E.-S.); by1401824@student.qu.edu.qa (B.A.-S.Y.)

**Keywords:** green adsorbents, green tea waste (GTW), wastewater treatment, methylene blue, full factorial design

## Abstract

Adsorptive removal of methylene blue (MB) from contaminated water samples was achieved using green tea waste (GTW). Adsorption of MB onto raw (RGTW) and thermally treated waste (TTGTW250–TTGTW500) was explored. The performance of the tested adsorbents was assessed in terms of percentage removal of MB (%R) and adsorption capacity (*qe*, mg/g). A full factorial design (FFD) was employed to optimize the adsorption of MB onto both RGTW and TTGTW500. Four factors were studied: pH, adsorbent dose (AD), dye concentration (DC), and contact time (CT). Value for %R of 96.58% and 98.07% were obtained using RGTW and TTGTW500, respectively. FT-IR and Raman analyses were used to study the surfaces of the prepared adsorbents, and the IR spectrum showed the existence of a variety of functionalities on the surfaces of both the RGTW and thermally treated samples. BET analysis showed the presence of mesopores and macropores in the case of RGTW and micropores in the case of thermally processed adsorbents. Equilibrium studies indicated that the Freundlich isotherm best described the adsorption of MB onto both adsorbents. The maximum adsorption capacity (*q_max_*) was found to be 68.28 and 69.01 mg/g for RGTW and TTGTW500, respectively, implying the superior capacity of TTGTW500 in removing MB. Adsorption of MB was found to proceed via chemisorption (RGTW) and physisorption (TTGTW500), as indicated by the Dubinin–Radushkevich (D-R) isotherm. A pseudo-second order (PSO) model best demonstrated the kinetics of the MB adsorption onto both adsorbents.

## 1. Introduction

Water pollution is becoming one of the most serious concerns that our world is confronting, if not the most serious of all. The increase in the number of the Earth’s inhabitants combined with incessant climate change and the consequent escalation of anthropogenic activities with constant but waning energy sources are the fundamental reasons behind this dilemma. With this fast progress of human life, numerous contaminants are threatening aquatic systems. Consequently, water pollution is becoming a critical health and environmental concern [1,2].

Various organic contaminants exist in the environment. As per the World Bank report, dyes are major contributors to water pollution [3,4]. Dyes can reach the water systems via different routes, e.g., from paper, textile, cosmetics, leather, plastics, and foodstuff industries. Among these routes, the tanning industry is one of the most important sources of aquatic systems’ continuous pollution. Approximately 5% of tanning wastes end up as effluents that are usually discarded into lakes, rivers, and ponds. Being colored and possessing variable chemical structures and different physicochemical properties, dyes represent a real burden on the ecosystem [3,4,5,6,7,8,9,10,11].

**Scheme 1:**
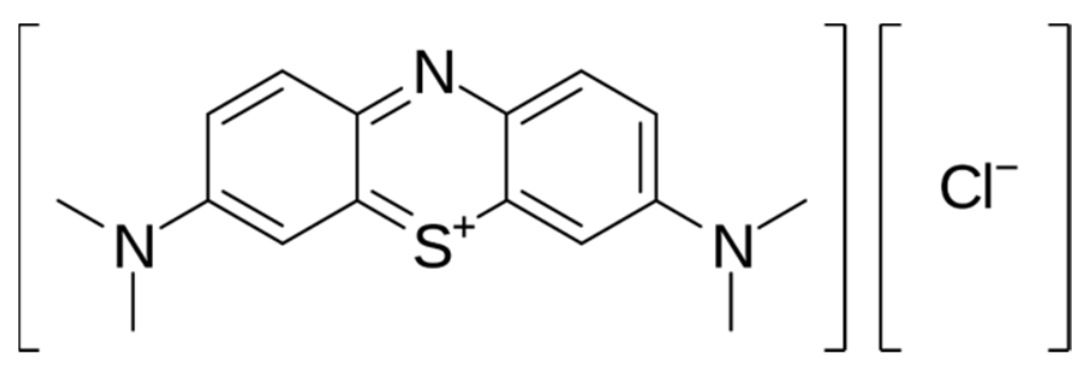
Chemical structure of methylene blue (MB).

One commonly used dye is methylene blue (MB), a cationic dye known as methylthioninium chloride (C_16_H_18_ClN_3_S; Scheme 1). MB is frequently used as a biological stain, e.g., in endoscopic polypectomies, for the injection into the submucosa surrounding the polyp to be removed; in chromoendoscopy; and to test the urinary tract for leaks. Moreover, MB is copiously released during the dyeing process in the textile industry. Reports show that MB produces toxic effects on the gastrointestinal system if ingested and an irritating effect on eyes in cases of contact [4,5,6].

Due to the associated health and environmental concerns, several remediation efforts have been reported to eliminate dyes from wastewater [3,4,5,6,7,8,9,10]. Among the reported techniques, adsorption is advantageous when compared to the other conventional approaches. Adsorption is a feasible, non-destructive technique with high removal efficiency, and it does not require prior treatment procedures and can be accomplished using eco-friendly materials with the lowest possible consumption of energy and resources [3,4,5,6,7,8,9,10,11,12,13].

Green adsorbents, a novel class of biosorbents, derived from agricultural/animal wastes are now commonly used for biosorption of contaminants from wastewater. Offering a compendium of advantages, including availability at low cost, potential for upcycling into value-added products for waste removal, a rich chemical composition with high surface area, and potential for modification, these wastes are progressively replacing the commercially available adsorbents [6,7,8,9,10,11,12,13].

In the same itinerary, the literature shows an escalating interest in the eco-structuring of green and eco-friendly biosorbents. The target is to boost the removal efficiency of the developed biosorbents by exerting control over the variables affecting the adsorption process. This control can be approached using multivariate analysis, where the process variables are changed simultaneously in a pre-designed scheme known as the factorial design. With the capacities to save time and resources, preserve method greenness, and produce high quality data, factorial designs are gradually replacing the traditional univariate analysis-based approach [14,15].

The green tea (GT) beverage is an infusion/decoction made from the leaves and buds of *Camellia sinensis* [16]. Tea is the one of the oldest beverages and the second most consumed beverage after water. Worldwide exports are dramatically escalating and expected to hit—as per the Food and Agriculture Organization of the United Nations (FAO) report—750,981 tones by 2023. This growth could be attributed to increased income per capita, especially in the major producing country, China. Being rich in active ingredients, e.g., tannins (with antibacterial and antioxidant properties), caffeine (helps eliminate stress), and vitamin B (promotes the secretion of digestive fluids and protects the mucous membranes), the extensive consumption of GT is usually associated with thoughts of health and fitness [17,18,19]. However, GT is abundantly available as household waste. Moreover, this waste is oxygen-demanding and degradation-resistant, an issue that needs a thorough look with regard to its impact on the environment when not properly eliminated.

In the current approach, green tea waste (GTW) was explored as an adsorbent for MB from wastewater samples. The structuring of this adsorbent was approached either by converting GTW into activated biochar through thermal treatment or by using the untreated GTW in a trial to develop a handy sorbent available in the household. Few approaches have been reported in the literature for the use of spent tea residues to remove dyes from wastewater samples, with more attention being paid to black tea. To the best of our knowledge, all the reported efforts were based on the conventional univariate approach where each variable is treated as a separate entity and only one factor is varied at a time. Table 1 shows a summary of these approaches, including the reported percentage removal (%R) as well as the adsorption capacity (*q_e_*, mg/g) [20,21,22,23,24].

As the objective of the current investigation was to structure a green, cost-effective, and efficient adsorbent via recycling of waste materials, a two-level full factorial design (2*^k^*-FFD) was executed. In this design, *k* denotes the number of independent variables to be investigated at the two levels. Four factors (pH, GTW adsorbent dose ‘AD’, the concentration of MB dye ‘DC’, and contact time ‘CT’) plus two responses (%R and *q_e_*) were assessed [14,15,25,26]. The performances of raw green tea waste (RGTW) and the thermally treated waste at 500 °C (TTGTW500) for MB removal were studied and optimized. Different kinetic and equilibrium models were used to assess the experimental data.

## 2. Experimental

### 2.1. Materials and Reagents

All chemicals and reagents were of analytical grade and were used as purchased. Ultrapure water (18.2 MΩ) was used to prepare and dilute dye solutions. Sodium tetraborate-10-hydrate (Na_2_B_4_O_7_·10H_2_O), sodium hydroxide, hydrochloric acid, and methylene blue were purchased from Sigma-Aldrich (St. Louis, MO, USA). The pH of methylene blue (MB) dye solutions was adjusted to the desired pH value using a mixture of 50 mM Na_2_B_4_O_7_·10H_2_O and 0.1 M NaOH or 0.1 M HCl. The color of the MB dye solution did not change over the studied pH range.

### 2.2. Instrumentation and Software

An Agilent diode-array UV-Vis spectrophotometer (Agilent, Santa Clara, CA, USA) with 10 mm quartz cells was used to measure the absorbance of samples prior to and after adsorption. A Jenway pH meter (Jenway, Staffordshire, UK) was used to measure the pH of tested samples. A Thermo Scientific centrifuge (ST8 Benchtop, Thermo Scientific, Waltham, MA, USA) was utilized to separate the adsorbent from the supernatant. Fourier-transform infrared spectroscopy (FT-IR, Bruker Alpha, Billerica, MA, USA) was used to identify the surface functional groups of the as-prepared GTW adsorbents. Similarly, the surface morphology was studied using scanning electron microscopy (SEM, FEI, Quanta 200, Thermo Scientific, Waltham, MA, USA) and energy-dispersive X-ray spectroscopy (EDX). The surface area, pore size, and volume of the GTW were determined using an ASAP 2020 accelerated surface area and porosimetry system (Micrometrics ^TM^, Norcross, GA, USA). The sample was initially degassed and then the N_2_ adsorption–desorption isotherms were collected at 77K, followed by the determination of the surface area using the Brunauer–Emmett–Teller (BET) equation. Additionally, t-plots and the Barrett–Joyner–Halenda (BJH) equation were used to determine the pore volume. The thermal stability of the raw green tea waste sample (RGTW) at a temperature range of 50–800 °C was investigated using a thermal gravimetric analyzer (TGA; PerkinElmer-TGA400, Waltham, MA, USA). The Raman spectrum (in the range of 50–3500 cm^–1^) was collected for the raw and the thermally treated samples using a Raman microscope (DXRTM 2; Thermo Scientific, Waltham, MA, USA), including a 532 nm laser beam and 10 mW power. Full factorial design (FFD) was undertaken by using Minitab^®^18 software purchased from Minitab Inc. (State College, PA, USA).

### 2.3. Preparation of the Tested Adsorbents

Green tea leaves were bought as loose tea from local supermarkets in Doha, Qatar. The obtained samples were mixed, boiled with distilled water for 15 min, and then filtered. This treatment was repeated ten times. The GTW was then dried in the oven for six consecutive days at 60 °C. The dried sample was ground using an electrical grinder and sieved using a 1 mm sieve. The obtained sample was named ‘raw green tea waste’ (RGTW). Portions of this sample were further thermally treated at different temperatures ranging from 250–500 °C, and the resultants were labeled ‘thermally treated green tea waste’ (TTGTW250–500).

### 2.4. Preparation of the Dye Samples

Water samples, artificially contaminated with methylene blue (MB), were prepared by dissolving the required amounts of MB in deionized water to a final concentration of 100 ppm stock solution. Further dilutions (10, 20, and 30 ppm) were prepared in the same solvent. The pH of the MB solutions was adjusted to the desired value using the borate–HCl or borate–NaOH mixtures. Three calibration curves for the MB at the selected pH levels (Table 2) were constructed by measuring different concentrations of MB at 666 nm.

### 2.5. Full Factorial Design (2^4^-FFD)

The examined variables and their boundaries are shown in Table 2. For both RGTW and TTGTW500, the selected design generated a total of twenty experiments, with sixteen runs in the base design plus four added central points (Ct Pt). Runs were conducted over two blocks with a resolution V within the block. The design structure is shown in Table 2 along with the measured (observed) as well as the predicted responses. Observed responses were calculated using Equations (1) and (2) as follows:

(1) $$\%R=\frac{C_{0}-C_{eq}}{C_{0}}\times 100$$

(2) $$q_{e}=\frac{C_{0\;}-C_{eq}}{w}\times V$$

where *C*_0_ (ppm) is the MB initial concentration, $C_{eq}$ (ppm) is the concentration of the MB dye at equilibrium, *V* (L) is the solution volume, and *W* (g) is the mass of adsorbent.

### 2.6. Point of Zero Charge (pH_PZC_)

The point of zero charge (pH_PZC_) for both RGTW and TTGTW500 samples was determined using the pH drift method [27]. Eight samples were prepared by adding 1.0 g of the adsorbent to each of eight Erlenmeyer flasks containing 50 mL of 0.01 M NaCl. The pH in each flask was adjusted to values ranging from 2.0 to 9.0 ± 0.2 using either 0.1 M HCl or 0.10 M NaOH, followed by shaking for 24 h in an automatic shaker at a speed of 150 rpm. The experiment was repeated three times. Eventually, the final pH of each solution was measured and then plotted against the initial pH. The pH_PZC_ was then determined from the intersection point of the obtained lines between the initial and the final pH.

### 2.7. Equilibrium and Kinetics Studies

Equilibrium studies were performed using a 1000 ppm stock solution of MB. Serial dilutions of the stock solution in the range of 40–400 ppm were prepared in the same solvent and the pH was adjusted to pH 9.0 ± 0.2 for RGTW and 5.0 ± 0.2 for TTGTW500 using the mentioned pH adjustment solution. Equal masses of RGTW and TTGTW500 (0.100 ± 0.005 g) were then added to 13.00 mL of the prepared solutions, which were shaken using a mechanical shaker at a steady speed of 150 rpm for 20 h and then filtered. The absorbance of the filtrate was measured at 666 nm. On the other hand, the kinetics studies were carried out for the studied adsorbents using a 200 mL MB dye solution (300 ppm, pH 9.0 ± 0.2 for RGTW and 30 ppm, pH 5.0 ± 0.2 for TTGTW500), and the mass of the adsorbent was 1.500 ± 0.005 g. One sample was taken at periods of around 1 min for a total time of 40 min.

## 3. Results and Discussion

### 3.1. Preliminary Screening of the Adsorption Performance

An initial screening of the adsorption performance of the five adsorbents was conducted. Assessment was based on finding the adsorbent with the best %R and *q_e_* (mg/g). The obtained results, shown in Table 3, reveal that RGTW had the highest adsorption efficiency for MB in terms of %R and *q_e_*. On the other hand, it was observed that the adsorption efficiency decreased as the temperature increased until the TTGTW300 sample. Adsorption efficiency started to increase again from TTGTW400 to TTGTW500. To analyze such behavior, different factors affecting the adsorption efficiency of MB onto both the RGTW and TTGTW500 samples are explained in the following sections along with an interpretation of these findings.

### 3.2. Design Analysis

#### 3.2.1. Quality Charts and Analysis of Variance (ANOVA) Testing

**Figure 1:**
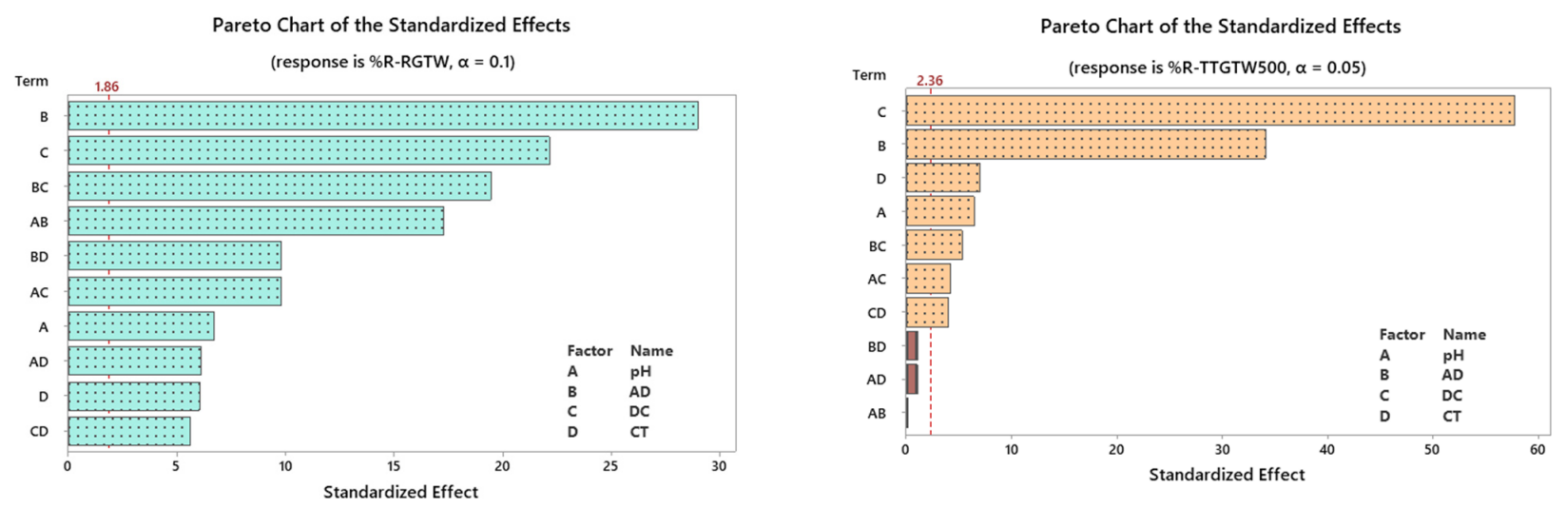

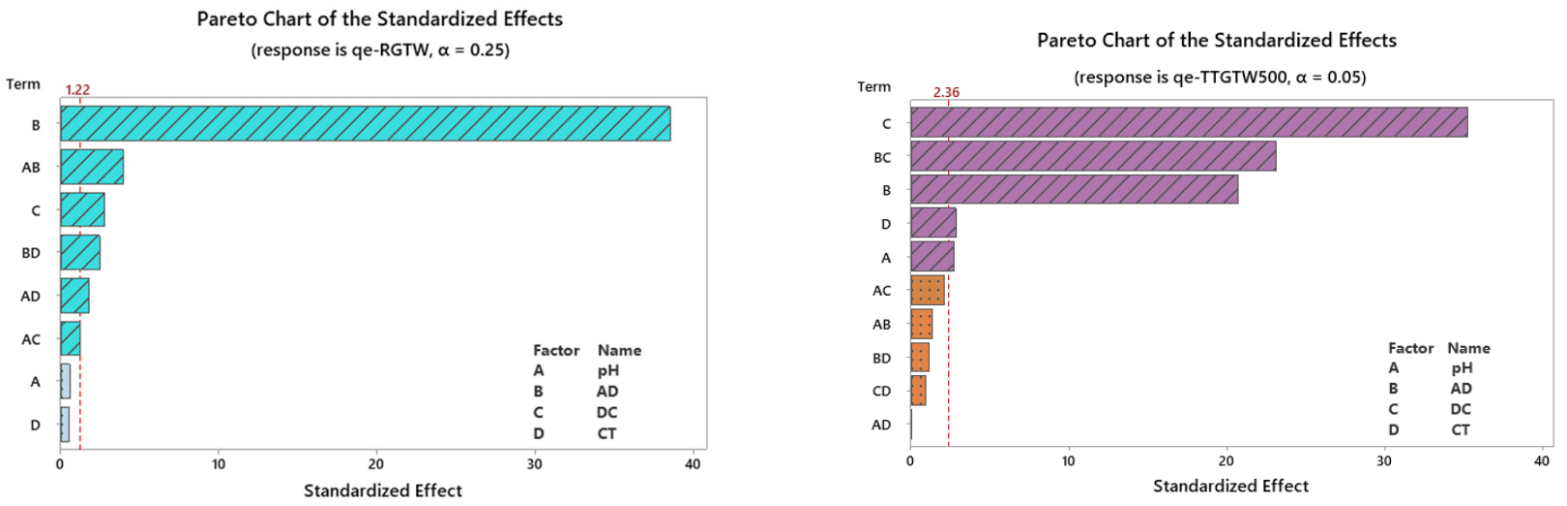
Pareto charts of standardized effects for the two responses using RGTW and TTGTW500 as adsorbents.

To test whether a variable was statistically significant and affected the measured response(s), a Pareto chart was employed (Figure 1). The impact of the variables affecting the removal efficiency of RGTW was different compared to the same response in the case of TTGTW500. For example, in the case of having the %R as the assessed response, the RGTW dose (AD, B) was the most statistically significant variable, as opposed to the concentration of MB (DC, C) in case of TTGTW500. Similar influences could be observed in case of *q_e_*. Since the Pareto chart does not show the direction of the response, other quality charts (normal and half-normal plots) were used (figures not shown). Analogous conclusions were drawn from the ANOVA testing, where variables having *p*-values less than 0.05 were considered statistically significant (table not shown). It is worth mentioning that the measured responses were obtained following a Box-Cox response transformation [28], either alone or together with stepwise analysis.

In the same context, the magnitude, and the direction of the impact of the variables on each response could be determined from the coefficients and the signs for each variable, respectively and as reflected in the regression models shown below. As shown in the Equations (3)–(6), for example, the pH seemed to have a higher magnitude in the case of the RGTW compared to the thermally processed sample. Moreover, the impact of pH on %R_RGTW_ was more notable compared to that on the *q_e_*_RGTW_. For the TTGTW500, however, the lowest %R and *qe* were obtained at pH 7.0, while at pH 5.0 and 9.0 both responses were maximum. Model summaries are shown in Table 4. Predicted responses (Pred.) were calculated using the generated models. As shown in Table 4, the values of the coefficient of determination, *R*^2^, were high enough to indicate model linearity. Also, the values of *R*^2^—adj were close to the values of *R*^2^—pred, implying that model could predict a new response efficiently. A comparison between the measured and the predicted responses using the values of relative error showed that the error ranged between 0 and 0.27, signifying the accuracy of the model.

(%R_RGTW_)^2^ = 6095 + 443.5 pH + 27.25 AD − 405.3 DC + 49.36 CT − 3.789 pH × AD + 21.54 pH × DC − 4.884 pH × CT + 0.8544 AD × DC − 0.1567 AD × CT + 0.899 DC × CT + 1692.1 Ct Pt, (3)

(−*q_e_*_RGTW_)^−2^ = − 0.0233 + 0.00659 pH − 0.000466 AD − 0.001533 DC + 0.000817 CT − 0.000043 pH × AD + 0.000134 pH × DC − 0.000070 pH × CT − 0.000002 AD × CT + 0.02639 Ct Pt, (4)

(%R_TTGTW500_)^3/4^ = 31.15 + 0.036 pH + 0.03360 AD − 0.8695 DC − 0.0365 CT + 0.000150 pH × AD − 0.03299 pH × DC + 0.00300 pH × CT + 0.000827 AD × DC + 0.000064 AD × CT + 0.002275 DC × CT + 1.780 Ct Pt, (5)

(*q_e_* _TTGTW500_) = 13.009 − 0.0153 pH − 0.04209 AD − 0.3806 DC + 0.00628 CT + 0.000411 pH × AD − 0.00628 pH × DC − 0.00005 pH × CT + 0.001384 AD × DC − 0.000025 AD × CT + 0.000200 DC × CT − 0.550 Ct Pt, (6)

#### 3.2.2. Contour and Surface Plots

**Figure 2:**
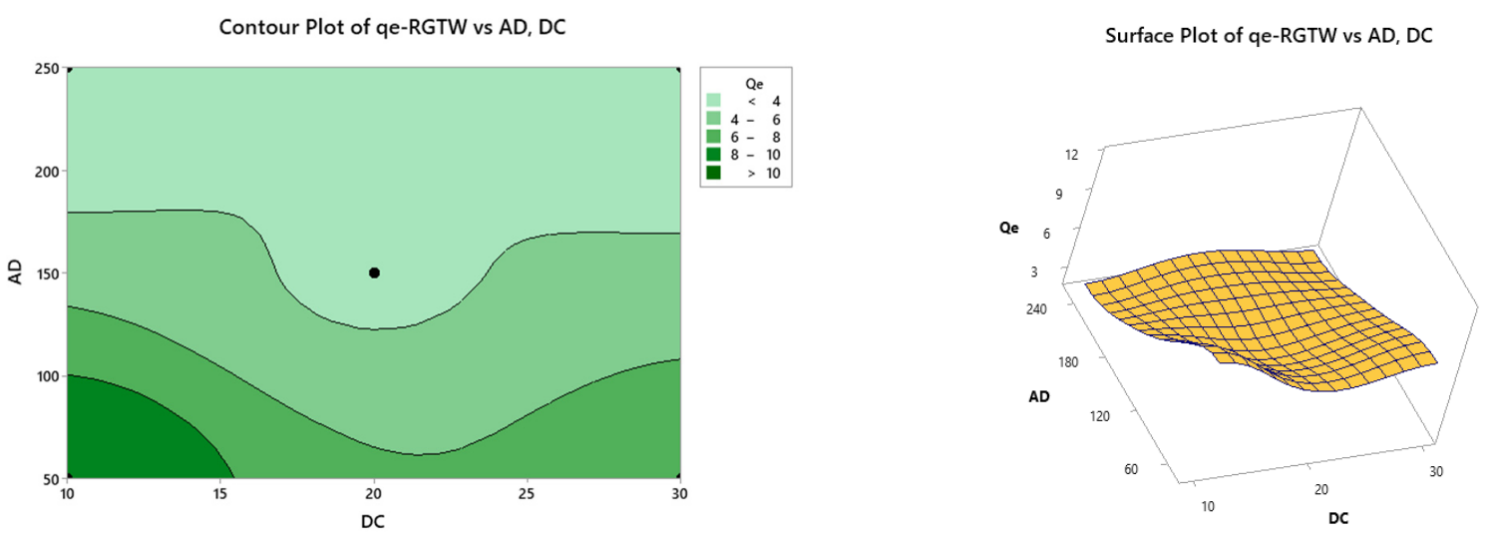
Sample contour (2D—**left panel**) and surface (3D—**right panel**) plots for the impact of DC–AD combination on *q_e_* using RGTW as adsorbent.

Two-dimensional (2D) and three-dimensional (3D) representations of data were used to assess the impact of variables on the measured response surface. As shown in Figure 2, 2D sample contour plots (left panel) were used to study the impact of a factorial combination AD–DC on the *q_e_* (mg/g) response surface using RGTW as adsorbent. As shown by the legend, the darkest green regions represent the regions with the maximum response and vice versa for the light green regions. Therefore, as shown by the contour plot, the adsorption capacity of RGTW could exceed 8 mg/g (up to 10 mg/g) using a combination of an AD of 50–100 mg/L and a DC of 10–15 ppm. Surface plots were used for the same job but in a 3D orientation. The maximum response is shown by the elevated ridge.

#### 3.2.3. Response Optimization

To obtain the optimum conditions that could maximize the measured responses, the desirability function (*d*, individual desirability) was utilized. The desirability of a certain factorial blends was decided based on the value of *d*, where the closer the *d*-value was to 1.000, the more favorable such a blend was considered to be. Table 5 shows a summary of the optimum conditions together with the obtained values of *d* for each response.

### 3.3. Characterization of GTW Samples

#### 3.3.1. FT-IR Analysis and Proposed Adsorption Mechanism

Leaves of green tea, like other biomasses, consist mainly of cell walls (comprised of lignin, cellulose, hemicellulose, proteins, condensed tannins, etc.). These constituents, in turn, are rich in functionalities (hydroxyl, carboxylate, etc.) that can contribute efficiently towards pollutants’ removal [21,29,30,31,32,33,34].

**Figure 3:**
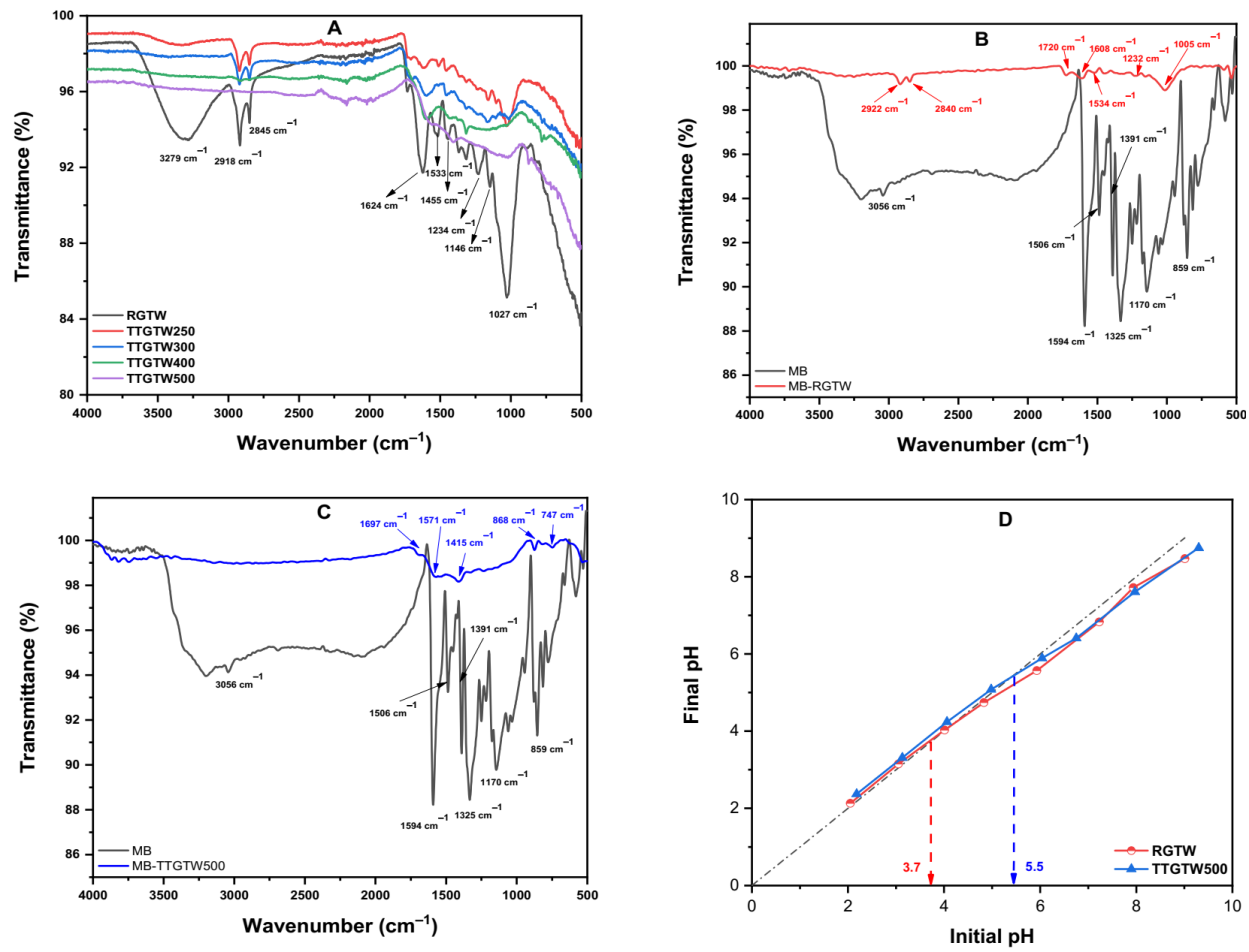
FT-IR spectra of (**A**) RGTW and the thermally treated samples (TTGTW250–TTGTW500), (**B**) MB and RGTW loaded with MB (MB-RGTW), (**C**) MB and TTGTW500 loaded with MB (MB-TTGTW500), and (**D**) point of zero charge (pH_PZC_) for RGTW and TTGTW500 adsorbents.

Figure 3A shows the FT-IR spectra of the RGTW, TTGTW250, TTGTW300, TTGTW400, and TTGTW500 samples. For RGTW, TTGTW250, and TTGTW300, the obtained spectra showed almost the same peaks but with a reduced intensity in the cases of TTGTW250 and 300, most likely because of the thermal treatment. The broad band at 3279 cm^−1^ could have been related to the stretching vibrations of bonded O-H or N-H groups (in RGTW with high intensity and in TTGTW300 with much lower intensity) [30,31]. The troughs at 2918 cm^−1^ and 2845 cm^−1^ could be attributable to the –C–H stretching of the alkane (aliphatic) functional groups [30]. The peak at 1624 cm^−1^ could be attributable to the –C=O stretching vibration of the alkene and aromatic functionalities [31]. In addition, other peaks were observed for secondary amine groups at 1533 cm^−1^ and N–H bending at 1455 cm^−1^ [32]. A small peak appeared at 1342 cm^−1^, which might have corresponded to the C–H or –CH_3_ bending, whereas the –SO_3_ stretching was located at 1234 cm^−1^ [30,32,33]. Also, the bands at 1146 and 1027 cm^−1^ could be attributable to the C−O and C=O groups, respectively [31,32,33]. On the other hand, the FT-IR spectra of the thermally activated samples at 400 and 500 °C showed the disappearance of the peak at 3279 cm^−1^, most likely because of the thermal treatment. The shifting of the other peaks to a lower intensity compared to that of RGTW TTGTW250 and 300 was observed, an issue that suggests the involvement of a different adsorption mechanism in the case of TTGTW500.

On the other hand, the IR spectrum of the free MB (Figure 3B,C) reflected the chemical structure of MB. The intense bands at 3056, 1594, 1506, and 859 cm^−1^ could have been related to the deformation vibrations in the condensed aromatic cycle. In addition, the band at 1170 cm^−1^ might be attributable to the presence of the C=C skeleton of the aromatic ring [35]. The spectra of both RGTW and TTGTW500 following the dye adsorption showed the presence of MB peaks with minor shifts and slightly different intensities. For instance, the band at 1506 cm^−1^ (Figure 3B) shifted by 28 cm^−1^ following the adsorption of MB onto the RGTW. These findings could help understanding the adsorption of the MB onto both adsorbents.

In parallel, RGTW showed a pH_PZC_ of 3.7 ± 0.2 compared to a value of 5.5 ± 0.2 in the case of TTGTW500 (Figure 3D). MB is a cationic dye with a pK_a_ value of 5.6 [36]. As per the FFD optimization findings, pH 5.0 is the optimum pH for a maximum %R. By and large, the degree of adsorption of charged dyes onto an adsorbent surface is essentially affected by the surface charge of the adsorbent, which in turn is controlled by the solution pH [36,37]. Therefore, at pH 5.0 (>pH_PZC_), the RGTW surface has a negative charge, while MB is positively charged (pH < pK_a_). Therefore, electrostatic interaction between MB and the negatively charged functionalities on the surface of RGTW is a possible reaction mechanism. When *q_e_* was the response measured, the pH was not statistically significant (Figure 2). For TTGTW500, the DC was the most statistically significant variable impacting both responses (Figure 2). In the same context, at pH 5.0 (almost equal to pH_PZC_ of TTGTW500), the surface of the TTGTW500 is neutral and, therefore, although pH 5.0 achieved the maximum response(s), the occurrence of electrostatic interaction might not be the best way to describe the adsorbent–adsorbate interaction in case of TTGTW500. As per the FT-IR data, functional moieties were almost absent on the surface of the thermally treated adsorbent. Therefore, π-π stacking between the π-system of the biochar and the aromatic system of MB is another possible mechanism [38]. Along with the mentioned routes for chemical interaction of the adsorbent with the adsorbate, the occurrence of physisorption cannot be excluded.

#### 3.3.2. Raman Analysis

**Figure 4:**
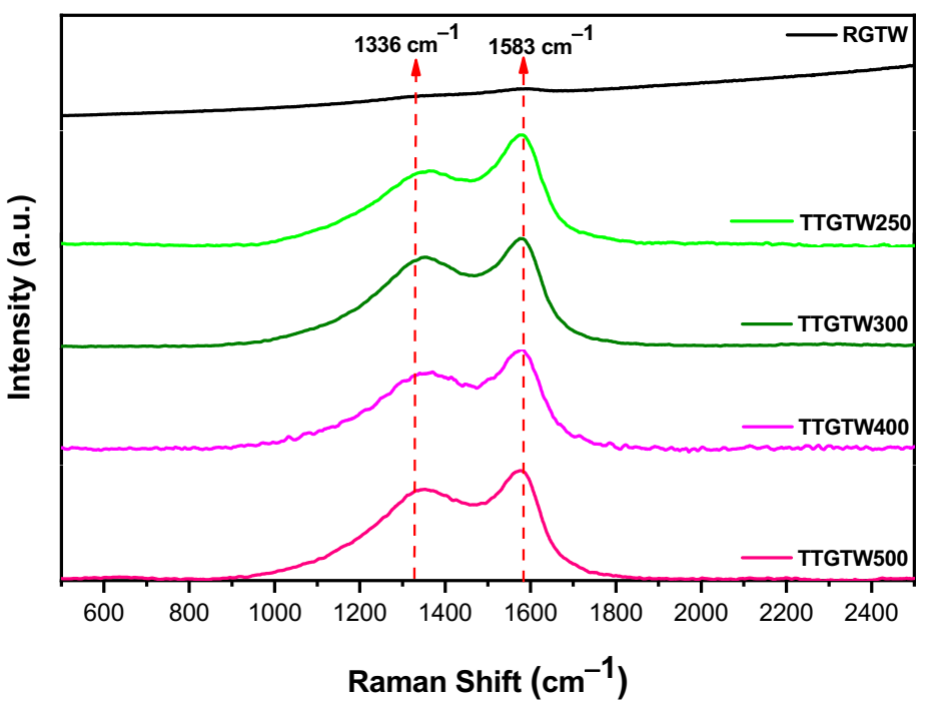
Raman analysis of RGTW and burnt samples at different temperatures.

The Raman spectra of RGTW and the thermally treated samples (250–500 °C) are shown in Figure 4. The spectrum of RGTW did not show any bands; on the other hand, the burnt samples showed a strong D-band appearing at 1351 cm^−1^ and a strong G-band at 1585 cm^−1^ [39,40]. The presence of these two peaks confirmed the decomposition of the organic matter and the formation of a carbonaceous material. The features of the carbon lattice, such as defects and sizes, can be presented by the D-band, but it does not explain the chemical structure of carbonaceous materials. Furthermore, the intensity ratio I_D_:I_G_ increased from TTGTW250 (0.66) to TTGTW500 (0.83). According to the obtained data, the thermal processing of the GTW samples increased the defect states in the *sp*^2^ plane of carbon, and this also proved the formation of the carbonaceous material following the burning process.

#### 3.3.3. CHN Analysis

Table 6 shows the data for the CHN analysis of RGTW and the thermally activated derived adsorbents (TTGTW250–500). The obtained data showed that the %C increased with thermal processing from 46.15 to 72.72% and this might be attributable to the conversion of RGTW into activated carbon, during which time the organic matter would have been destroyed by the thermal treatment, with consequent conversion into a carbonaceous material. On the other hand, the hydrogen concentration decreased from 6.4% in the RGTW to 2.95% in TTGTW500, which could be attributable to the loss of hydrogen during the heating process through water evaporation. The nitrogen concentration, in contrast, increased from 4.09% in the RGTW to 6.38% in the TTGTW500 sample.

#### 3.3.4. TGA Analysis of RGTW

**Figure 5:**
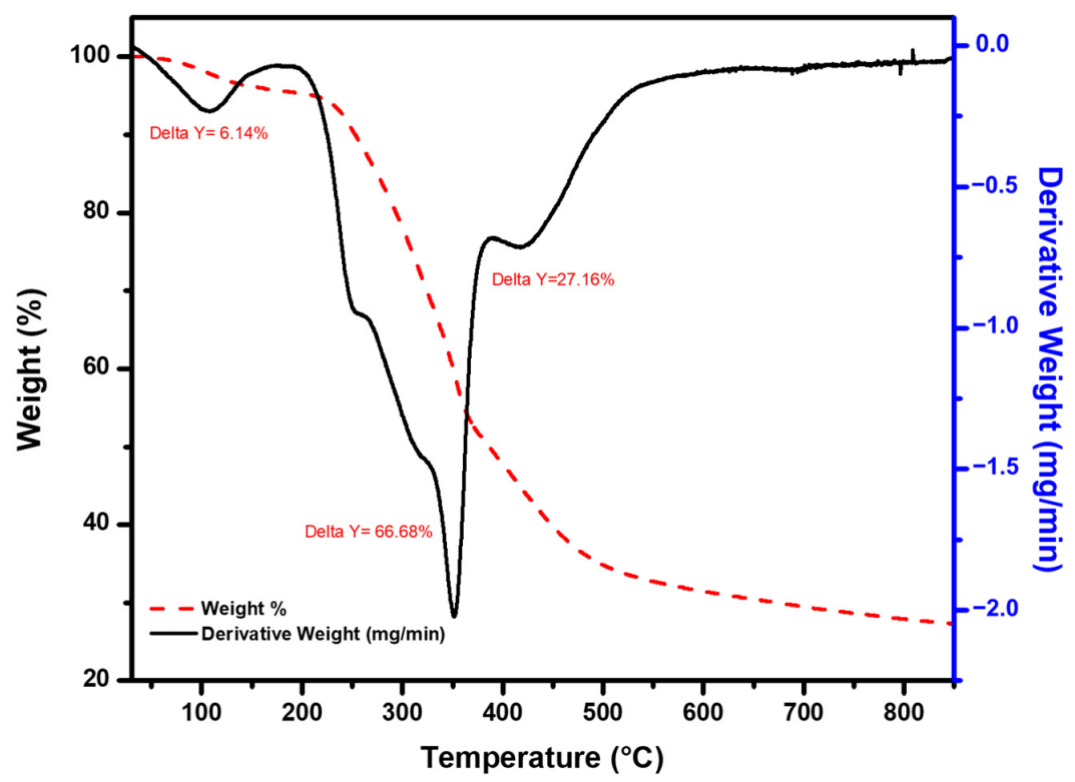
TGA analysis of the RGTW sample.

Thermogravimetric analysis of RGTW sample was performed under N_2_ and with a heating rate of 10 °C/min. The data represented in Figure 5 reveal that the weight loss for the RGTW sample occurred through two main steps as follows:

1. In the temperature range from 25 to 100 °C, the adsorbed water molecules were lost, followed by the loss of crystalline water at ~200 °C, which represented 6.14% of the sample;
2. In this step, >80% of the sample was decomposed between 200 and 600 °C; two major peaks at 350.97 °C could be observed in addition to three shoulders at 250.68, 313.97, and 421.40 °C, which could be attributable to the decomposition of the organic materials present in RGTW and the conversion to carbonaceous material.

#### 3.3.5. SEM Analysis

**Figure 6:**
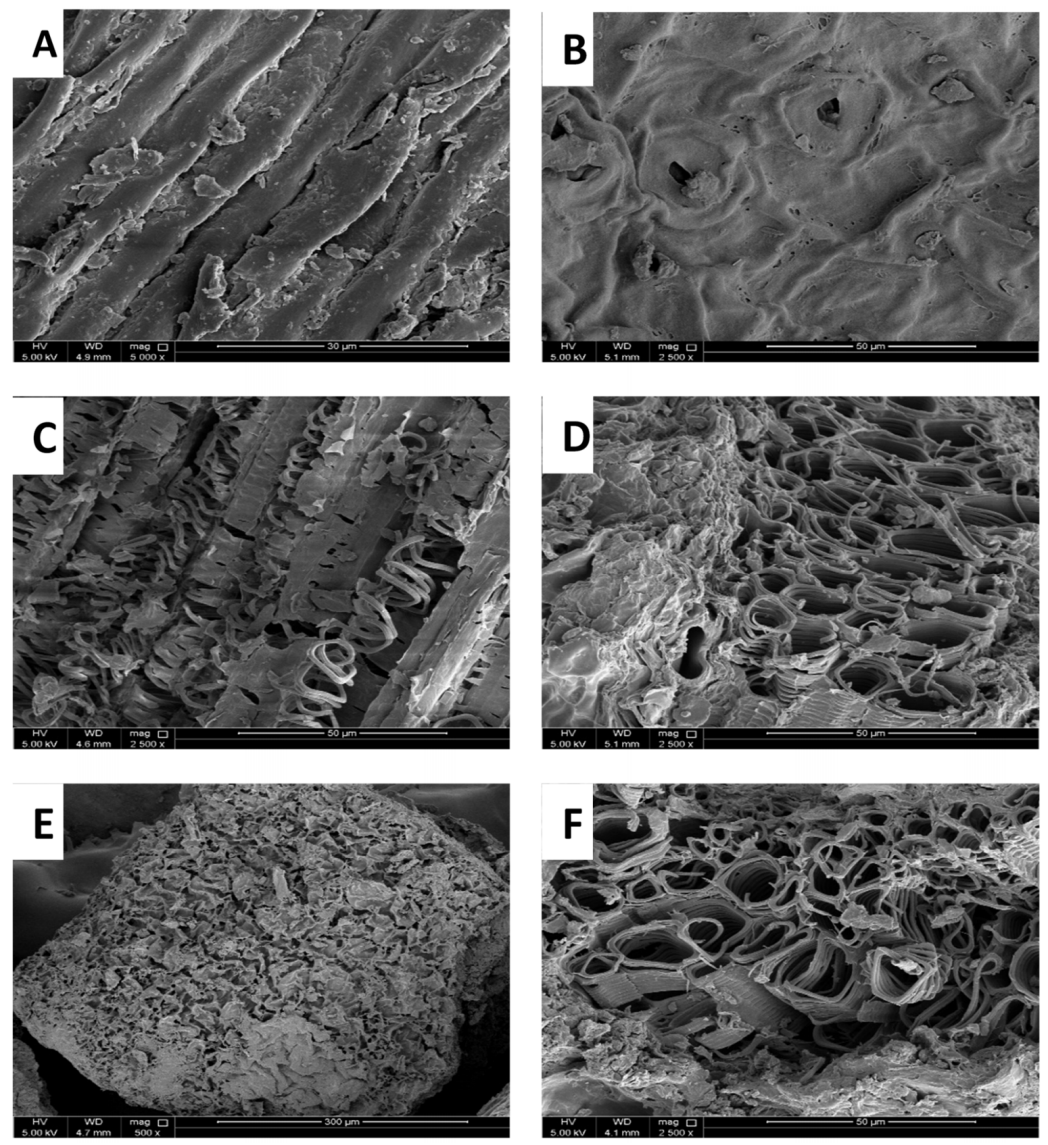
SEM micrographs of (**A**) RGTW, (**B**) TTGTW250, (**C**) TTGTW300, (**D**) TTGTW400, (**E**) TTGTW500, and (**F**) TTGTW500.

The surface morphologies of RGTW and the thermally treated samples were studied using SEM. The micrographs shown in Figure 6 illustrate the presence of a plain regular surface without any pores for both RGTW and TTGTW250. On the other hand, the micrographs of TTGTW300 show the development and the appearance of some pores. This finding could be attributable to the thermal decomposition of the upper layer of the tea leaf where the veins start to appear as columns inside the leaf. For the TTGTW400 and 500, the leaf veins appeared as long columns with large pores, and this could have provided a considerable surface outside and inside these columns, which could have affected the adsorption efficiency positively.

#### 3.3.6. BET Analysis

**Figure 7:**
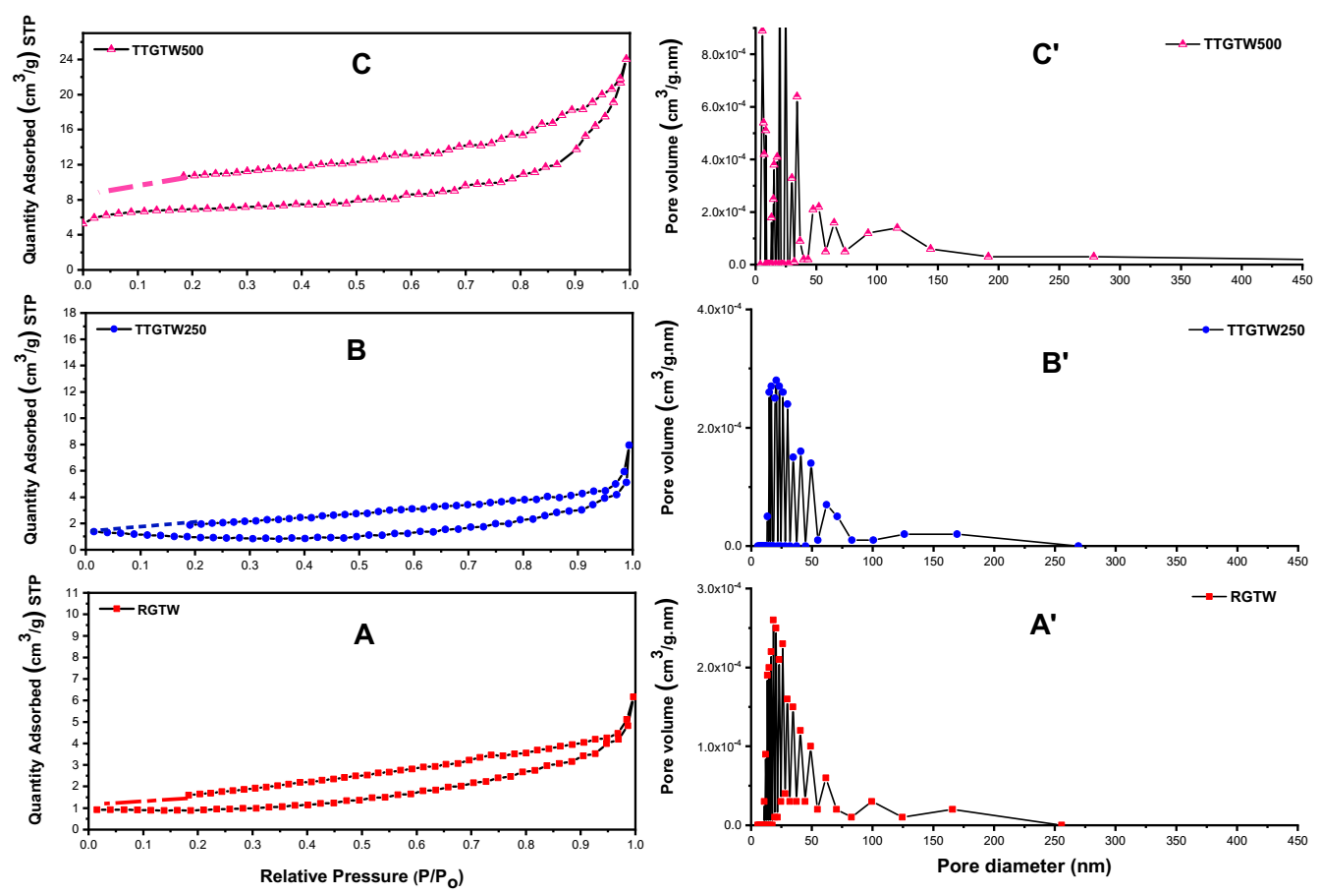
Nitrogen adsorption–desorption isotherms at 77K for RGTW (**A**), TTGTW250 (**B**), and TTGTW500 (**C**) and the relation between pore volume and diameter for RGTW (**A’**), TTGTW250 (**B’**), and TTGTW500 (**C’**).

**Figure 8:**
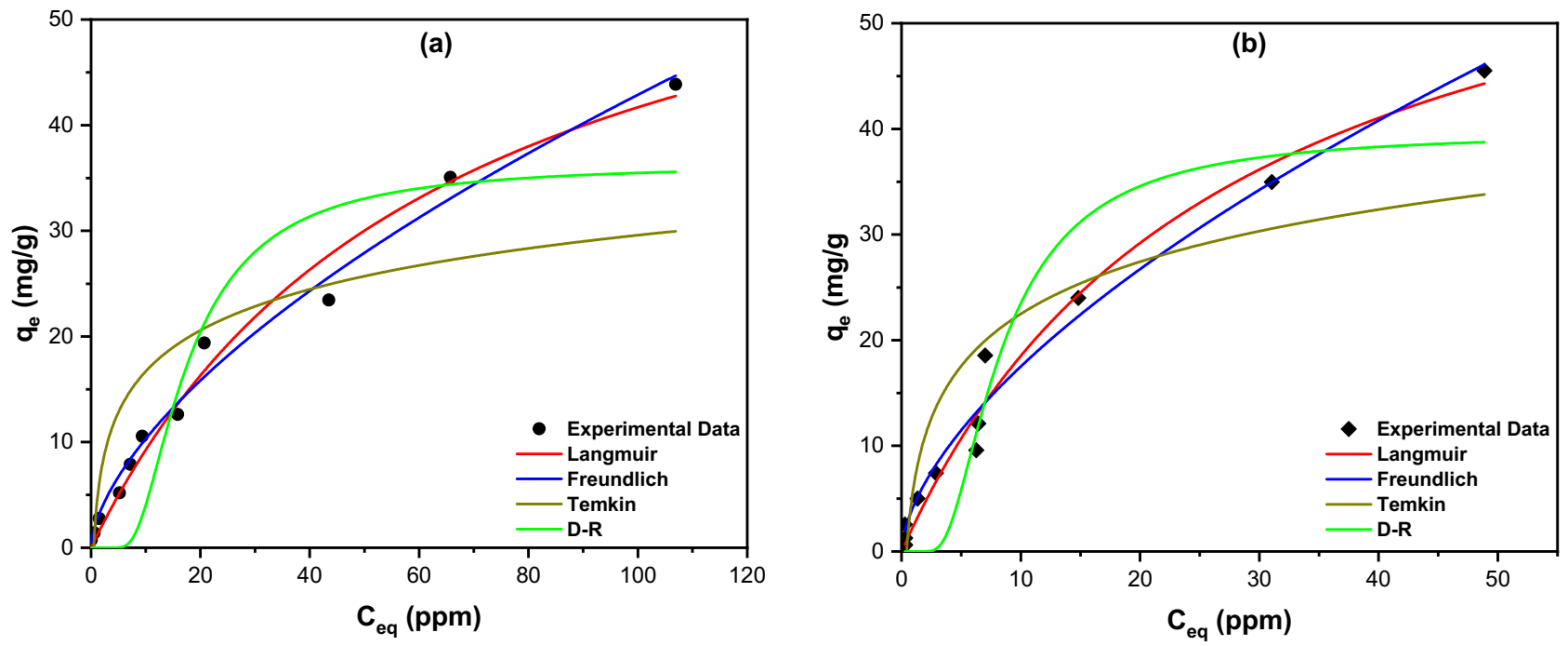
Isotherms for the adsorption of MB onto RGTW (**a**) and TTGTW500 (**b**). Four isotherms were investigated: Langmuir, Freundlich, Temkin, and Dubinin–Radushkevich (D-R).

The surface areas (SAs) of the as-prepared adsorbents were measured as shown in Table 7 and Figure 7. The obtained data show that the Langmuir SA increased from 3.84 m^2^/g for RGTW to 30.705 m^2^/g for TTGTW500. This increase in the SA could have been related to the release of the volatile materials and the formation of the large columns (vascular bundles) during the thermal treatment, as shown in the SEM micrographs. This finding provides evidence of the increased uptake of MB by TTGTW500 compared to TTGTW300. Moreover, the total pore volume increased from 0.0096 to 0.037 cm^3^/g as the pyrolysis temperature increased. Nonetheless, a decrease in average pore radius could be observed by increasing the pyrolysis temperature. This observation could have been due to the collapse of the rupture of the matrix of RGTW creating new micropores, an issue that may have facilitated the increase in the total pore volume and the decrease in the pore size. Based on these data, the thermal treatment of GTW increased the SA of the adsorbent, which could have had a great effect on its removal efficiency. The effects of the burning process on the pore volume and pore size can be easily verified in the SEM micrographs. Furthermore, Figure 7 shows that the three adsorbents exhibited a type III adsorption isotherm with an H3 hysteresis loop. This type of hysteresis loops indicates the aggregation of plate-like particles forming slit-like pores in loose assemblies. Moreover, Figure 8 also shows the presence of two main types of pores, mesopores (2–50 nm diameter) and macropores (>50 nm diameter), in RGTW and TTGTW250 and the appearance of a third type, micropores (<2 nm diameter), in TTGTW500 [41].

### 3.4. Equilibrium and Kinetics Studies of the Adsorption of MB onto RGTW and TTGTW500

The adsorption efficiency of the RGTW and TTGTW500 for MB was evaluated and equilibrium and kinetics studies were conducted on the two adsorbents. These studies included four adsorption isotherms, which are useful for determining the maximum adsorption capacity and the type of adsorption on the surface of the adsorbent biomass. Moreover, the type of interaction (chemisorption or physisorption) between both the adsorbates and the adsorbents’ surfaces could be determined based on the findings of these studies. Kinetics studies can give useful information about the type of adsorption process and whether it is pseudo-first order (PFO) or pseudo-second order (PSO). Furthermore, it can be used to determine the rate of adsorption, the thickness of the layer formed around the sorbent surface, the mechanism of the adsorption process, and whether it is controlled by adsorption or diffusion mechanisms.

#### 3.4.1. Equilibrium Studies

The relationship between the degree of accumulation of the adsorbed dye particles on the adsorbent surface and the concentration of adsorbate at a constant temperature can be expressed using the adsorption isotherms. The adsorption of MB onto the RGTW and TTGTW500 samples was investigated using four models: the Langmuir, Temkin, Freundlich, and Dubinin–Radushkevich (D-R) isotherms [42,43,44,45]. Single-layer homogeneous adsorption onto a biomass surface can be explained by using the Langmuir isotherm as shown in Figure 8a,b and Table 8. The Langmuir parameters can be determined by using the Langmuir equation, as follows:

(7) $$q_{e}=\frac{q_{m}\;K_{L}\;C_{eq}}{1+K_{L}\;C_{eq}}$$

where *q_m_* is the monolayer adsorption capacity and *K_L_* is the Langmuir equilibrium coefficient. Furthermore, the dimensionless equation also can be used to define the Langmuir equation:

(8) $$R_{L}=\frac{1}{1+K_{L}\;C_{0}}$$

where *R_L_* and $C_{0}$ (ppm) are the separation factor and the dye initial concentration, respectively. The *R_L_* value determines the adsorption favorability. Therefore, if *R_L_* is >1, the adsorption process is unfavorable; if *R_L_* = 1, the adsorption process is linear; if the value of *R_L_* is between 0 and 1, the adsorption is favorable and occurs spontaneously; but if *R_L_* = 0, adsorption is irreversible. The calculated *R_L_* value in the current investigation was <1 and >0, indicating that the adsorption of the MB onto both RGTW and TTGTW500 was spontaneous. In addition, the maximum adsorption capacity (*q_max_*) was found to be 68.28 and 69.01 mg/g for RGTW and TTGTW500, respectively. The obtained data show that the values of *q_max_* for the thermally treated samples were higher than for RGTW, an issue which could have been related to the larger SA of the TTGTW500 compared to RGTW.

The Freundlich isotherm is used to describe heterogeneous surface energies and is given by the following equation:

(9) $$q_{e}=K_{F}C_{eq}^{\frac{1}{n}}$$

In the above equation, *C_eq_* (ppm) represents the dye equilibrium concentration, *q_e_* (mg/g) is the amount of MB adsorbed/unit mass, and *K_F_* (mole·g^−1^) (L·mole^−1^)^1/n^ and 1/n are Freundlich coefficients (Figure 8a,b and Table 8). The Freundlich plot for the RGTW sample (Figure 8a) showed a good fit, with a coefficient of determination of 0.9878 and 1/n = 0.619. These findings confirm that the Freundlich isotherm was more applicable for the study of the adsorption of MB onto RGTW. The same behavior was obtained for TTGTW500 (Figure 8b), showing a good fit with *R*^2^ = 0.9809 and 1/n = 0.610.

The Temkin isotherm for the two studied adsorbents (Figure 8a,b) was used to gain insight into the interaction between MB and the adsorbent by determining the factor that described the fact that the heat of the adsorption of all the molecules in the layer decreased linearly with the adsorbent–adsorbate interactions. The data presented in Table 8 show that the *R*^2^ values were 0.7139 for RGTW and 0.8108 for TTGTW500, indicated that this isotherm was not applicable in the current study.

The D-R isotherm (Figure 8a,b) is usually employed to identify the type of the mechanism of adsorption onto a heterogeneous surface. The adsorption process can be classified into two mechanisms based on the value of the free energy: physical adsorption (free energy <8.0 kJ/mol) and chemical adsorption (when free energy >8.0 kJ/mol).

The obtained data, shown in Table 8, indicated the presence of two different adsorption mechanisms. Starting with RGTW, the free energy for the adsorption of MB onto RGTW was 8.45 kJ, implying the occurrence of chemisorption. This finding provides further support for the proposed adsorption mechanism discussed in the FT-IR section. On the other hand, the free energy for the adsorption of MB onto TTGTW500 was 5.27 kJ, indicating that the adsorption of MB in this case was physisorption. This could have been related to the SA of the adsorbent, as indicated by the SEM and BET analyses.

#### 3.4.2. Kinetics Studies

**Figure 9:**
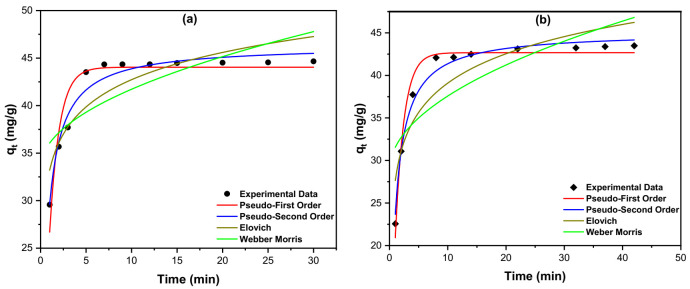
Kinetic models for the adsorption of MB onto RGTW (**a**) and TTGTW500 (**b**), including pseudo-first-order, pseudo-second-order, intraparticle diffusion (Weber–Morris), and Elovich models.

The kinetics of the adsorption of MB onto both RGTW and TTGTW500 were investigated by studying four kinetic models, including pseudo-first order (PFO), pseudo-second order (PSO), Elovich, and Weber–Morris (W-M) models [46,47,48]. The determined parameters for the PFO and PSO models, based on Figure 9a,b, are listed in Table 9. By comparing the values of the *R*^2^ of the two models, it was concluded that the experimental data were in good agreement with the PSO. Consequently, the reaction of the GTW samples with MB could be represented as follows:

(10) $$MB+GTW\;\overset{k}{\to }\;\left\{MB-GTW\right\}$$

The rate of the reaction, therefore, equaled *k* [MB] · [GTW], indicating that the adsorption rate depended on the adsorbate and the adsorbent.

The Weber–Morris (W-M) intraparticle diffusion model (Figure 9a,b) highlighted significant findings regarding the mechanism that controlled the MB diffusion for both adsorbents. According to the calculated data in Table 9, the adsorption reaction had a high intraparticle diffusion rate (2.62 mg·g^−1^·min^−0.5^) with a low boundary layer thickness (33.44 mg/g) for RGTW. On the other hand, TTGTW500 showed a higher intraparticle diffusion rate (2.77 mg·g^−1^·min^−0.5^) and a higher boundary layer thickness (38.76 mg/g) compared to the RGTW sample. The obtained data showed two different adsorption mechanisms depending on the type of adsorbent. For RGTW, as stated before, is the mechanism was chemisorption and mainly depended on the functional groups present on the adsorbent surface, leading to the formation of one layer on the surface and a low boundary layer thickness, confirming the findings from the D-R isotherm. On the other hand, the type of adsorption in TTGTW500 was physisorption (D-R isotherm), and it was mainly controlled by the presence of a large surface area in the thermally treated sample.

The Elovich model is commonly used to predict sorption mechanisms. As shown in Figure 9a,b, the high *R*^2^ values for the RGTW (0.792) and for the TTGTW500 (0.824) samples indicated that this model was not applicable for the adsorption of MB onto both adsorbents. Similarly, the model indicated that the initial sorption rate was higher than the desorption rate for both adsorbents. The initial sorption concentration rate (α) in the case of RGTW was 1.27 × 10^4^, compared to 1.28 × 10^22^ mg·g^−1^·min^−1^ in the case of TTGTW500.

## 4. Conclusions

The effectiveness of GTW as an adsorbent on clean wastewater samples from a hazardous dye, MB, was explored. A comparison between five adsorbents, RGTW and four thermally treated adsorbents (TTGTW250–500), was undertaken, and assessment was based on the %R and *q_e_*. To investigate how to reduce consumption of chemicals and resources and minimize the amount of waste, experiments were designed using full factorial design (FFD) as a platform. The objective was to achieve the highest %R and *q_e_* via the exertion of control over the process variables. A Pareto chart showed that the RGTW dose (AD) was the more significant variable compared to the concentration of MB (DC) in the case of TTGTW500. Similar conclusions were obtained with ANOVA at a 95.0 confidence interval (95.0 CI). In this context, both RGTW and TTGTW500 were demonstrated to achieve excellent removal for MB (96.58% and 98.07%, respectively). Different characterization techniques were used to explore the surface properties of the tested adsorbents. FT-IR analysis showed that the surface of the RGTW was rich in functional moieties, the intensity of which decreased with the increase in the temperature of the thermal treatment. Raman analysis confirmed the conversion of the adsorbent matrix into biochar with thermal processing. SEM and BET analyses showed a smooth surface in the case of RGTW and a porous surface with mainly meso- and macropores in the case of the thermally treated samples. Equilibrium studies further confirmed the characterization findings, and the occurrence of chemisorption in the case of RGTW and physisorption in the case of TTGTW500 were indicated by the D-R isotherm. It is worth mentioning that the value of *q_max_* was found to be 68.28 and 69.01 mg/g for RGTW and TTGTW500, respectively. Kinetics studies showed that adsorption followed a pseudo-second order kinetics for the two adsorbents. Although the TTGTW500 possessed a slightly higher adsorptive capacity compared to RGTW, the use of RGTW as an adsorbent could be more economic in terms of saving energy and the overall cost.

## Figures and Tables

**Table 1:** GTW as an adsorbent for dyes from wastewater.

Adsorbent	Dye(s)	Design of the Experiment	Kinetic Model	Isotherm Model	Surface Area (m^2^/g)	*q_e_*/*q_max_* (mg/g)	%R	Ref
Raw green tea waste (RGTW) Thermally treated green tea waste at 500 °C (TTGTW500)	Methylene blue (MB)	Multivariate analysis, 2*^k^*-FFD	PSO *	Freundlich	3.84 30.71	68.28 69.01	96.58 98.07	Current approach
Green tea dredge (carbonized green tea waste (CGT))	Remazol brilliant yellow	Univariate analysis	PSO *	Langmuir	ND *	40.65	ND **	[20]
Green tea leaf powder (GTLP)	Raw wastewater (90–95% reactive dye, 5–10% dispersive dyes)	Univariate analysis	ND **	Modified Freundlich isotherm and intraparticle diffusion	1.99	775 ADMI g^−1^ ***	ND **	[21]
Superparamagnetic Fe_3_O_4_ nanoparticles coated with green tea polyphenols (GTP): (Fe_3_O_4_@GTPs NPs)	Methylene blue	Univariate analysis	PSO *	Langmuir	126.79	7.25	95%	[22]
Waste green tea (WGT), untreated Six chemical treatments were used (four acidic—H_3_PO_4_, H_2_SO_4_, HCl, and tartaric acid—one oxidant—H_2_O_2_—and one basic—NaOH)	Malachite green	Univariate analysis	ND **	ND **	ND **	ND **	89–95%	[23]
Iron-based nanoparticles with extract from green tea (GTFe) Superparamagnetic iron oxide nanoparticles (smGT) derived from GTFe	Malachite green	Univariate analysis	ND **	ND **	ND **	ND **	93%	[24]

* PSO: pseudo-second order, ** ND: not determined, *** ADMI: American Dye Manufacturers’ Institute.

**Table 2:** Design matrix for a 2^4^-FFD prepared for the usage of RGTW and TTGTW500 for removal of MB from contaminated water samples. Responses are the %R and *q_e_* (mg/g). Following the application of the portrayed scenario, solutions were centrifuged at 4000 rpm for 10 min and then the absorbance of the supernatant was measured.

Independent Factors						Levels							
						−1, Low		0, Ct Pt			+1, High		
pH (pH, A, pH unit)						5.00		7.00			9.00		
Adsorbent (GTW) dose (AD, B, mg/50 mL)						50		150			250		
MB dye concentration (DC, C, ppm)						10		20			30		
Contact time (CT, D, min)						5		32.5			60		
**Run**	* Blk	**Variables**				**RGTW**				**TTGTW500**			
						**%R**		***q_e_***		**%R**		***q_e_***	
		**pH**	**AD**	**DC**	**CT**	**Obs.**	**Pred.**	**Obs.**	**Pred.**	**Obs.**	**Pred.**	**Obs.**	**Pred.**
01	1	+	+	+	−	90.79	91.67	2.26	2.31	25.07	25.63	0.63	0.56
02	1	0	0	0	0	92.90	93.73	3.91	3.63	52.30	51.80	2.40	2.33
03	1	+	+	−	+	82.52	83.10	2.24	2.25	95.26	96.67	2.38	2.45
04	1	+	−	−	−	89.66	89.93	11.06	10.40	58.84	58.37	7.33	7.30
05	1	−	+	−	−	96.53	97.58	2.41	2.43	96.02	94.17	2.40	2.27
06	1	−	−	−	+	88.32	87.99	10.97	9.73	62.73	63.04	7.74	7.91
07	1	0	0	0	0	94.60	93.73	3.50	3.63	53.40	51.80	2.60	2.33
08	1	+	−	+	+	79.09	78.44	9.89	9.72	3.20	4.58	0.31	0.55
09	1	−	−	+	−	36.00	36.07	4.27	4.55	5.33	5.20	0.67	0.70
10	1	−	+	+	+	92.62	92.89	2.31	2.31	46.09	47.47	1.15	1.29
11	2	+	+	+	+	89.89	89.59	2.24	2.21	40.24	37.96	1.00	0.87
12	2	−	+	+	−	89.94	89.05	2.36	2.32	36.08	36.41	0.91	0.97
13	2	+	+	−	−	92.05	90.94	2.38	2.35	89.76	90.42	2.24	2.37
14	2	+	−	+	−	69.34	69.31	8.58	7.97	0.000	0.000	0.00	0.00
15	2	−	−	−	−	79.74	79.43	5.99	5.67	60.65	62.19	7.51	7.54
16	2	0	0	0	0	94.01	93.73	3.91	3.63	51.05	51.80	2.12	2.33
17	2	0	0	0	0	93.40	93.73	3.30	3.63	50.45	51.80	2.20	2.33
18	2	−	−	+	+	60.19	61.03	6.11	5.10	12.19	11.12	1.52	1.29
19	2	−	+	−	+	96.58	96.08	2.41	2.43	98.07	97.86	2.44	2.37
20	2	+	−	−	+	91.61	91.92	11.41	15.63	62.91	61.53	7.84	7.66

* Blk: block, Obs.: observed, Pred.: predicted.

**Table 3:** A comparison of the adsorption aptitudes of the five adsorbents in terms of %R and *q_e_* using an AD = 200 mg/50 mL, pH = 7.0 ± 0.2, CT = 30 min, and DC = 30 ppm. The responses shown were calculated using Equations (1) and (2).

Adsorbent	%R	*q_e_* (mg/g)
RGTW	93.05	2.41
TTGTW250	87.28	2.26
TTGTW300	28.16	0.73
TTGTW400	35.77	0.87
TTGTW500	57.90	1.50

**Table 4:** Summary of the regression models.

*R*^2^ %	*R*^2^—adj %	*R*^2^—pred %
99.70	99.28	97.51
99.36	98.78	98.18
99.85	99.59	98.24
99.69	99.16	96.97

**Table 5:** Optimum conditions for maximizing the responses together with the obtained *d* values.

Optimum Conditions				Maximum %R	Maximum *q_e_* (mg/g)	*d*-Value
pH	AD (mg/50 mL)	DC (ppm)	CT (min)			
RGTW						
5.0	250.0	10.0	5.0	97.58		1.0000
9.0	50.0	10.0	60.0		15.63	1.0000
TTGTW500						
5.0	250.0	10.0	60.0	97.86		0.9979
5.0	50.0	10.0	60.0		7.91	1.0000

**Table 6:** CHN analysis of RGTW and TTGTW prepared at different temperatures.

Sample Code	N (%)	C (%)	H (%)
RGTW	4.094	46.151	6.435
TTGTW250	5.694	55.885	6.287
TTGTW300	5.958	60.186	5.138
TTGTW400	5.253	64.551	3.890
TTGTW500	6.378	72.723	2.952

**Table 7:** BET analysis of RGTW and thermally treated green tea samples.

Parameters	RGTW	TTGTW250	TTGTW300	TTGTW400	TTGTW500
Langmuir SA (m^2^/g)	3.84	4.51	6.61	8.94	30.71
Total pore volume (cm^3^/g)	0.0096	0.0113	0.0124	0.017	0.037
Average pore radius (°A)	74.2	87.5	54.0	41.2	33.7

**Table 8:** Nonlinear equations and parameters of the Langmuir, Freundlich, Temkin, and Dubinin–Radushkevich isotherms for the adsorption of MB onto the RGTW and TTGTW500 samples.

Isotherm	Equations (Nonlinear Forms)	Parameters	RGTW	TTGTW500
Langmuir	$q_{e}=\frac{q_{m}\;K_{L}\;C_{eq}}{1+K_{L}\;C_{eq}}$	$q_{m}$ (mg/g)	68.28	69.01
		$K_{L}$ (L·mole^−1^)	0.0157	0.0367
		*R* ^2^	0.9833	0.9796
Freundlich	$q_{e}=K_{F}C_{eq}^{\frac{1}{n}}$	1n	0.619	0.610
		$K_{F}$ (mole/g)(L/mole)^1/n^	2.476	4.298
		*R* ^2^	0.9878	0.9809
Temkin	$q_{e}=\frac{RT}{b_{T}}\;\ln\left(A_{T}\;C_{eq}\right)$	$b_{T}$ (J/mole)	442.3	347.6
		$A_{T}$ (L/mole)	1.969	2.347
		*R* ^2^	0.7139	0.8108
D-R	$q_{e}=q_{s\;}\;$. exp (−β.$\;\epsilon^{2}$)	β	7 × 10^−9^	1.8 × 10^−8^
	$\epsilon =RT\left(1+\frac{1}{C_{eq}}\right)$	E (kJ/mole)	8.45	5.27
		$q_{m}$ (mg/g)	36.34	39.67
	E=12β	*R* ^2^	0.8535	0.9041

**Table 9:** The kinetics study results corresponding to Figure 9.

Model	Parameter	RGTW	TTGTW500
Pseudo-first order (PFO) $\frac{dq_{t}}{dt}$ *= k*_1_(*q_e−_q_t_*)	*K*_1_ (min^−1^)	0.931	0.674
	*q_e_* (mg/g)	44.04	42.66
	*R* ^2^	0.9011	0.9788
Pseudo-second order (PSO) $\frac{dq_{t}}{dt}$ = *k*_2_(*q_e_−q_t_*)^2^ where *K*_2_ is the rate constant (g·mg^−1^·min^−1^)	*K*_2_ (g·mg^−1^·min^−1^)	0.038	0.024
	*q_e_* (mg/g)	46.35	45.12
	*R* ^2^	0.9619	0.9861
Elovich model *q_t_* = $\frac{1}{\beta }\times ln\left(1+\alpha \beta t\right)$ where *q*_t_ is the adsorbed quantity at time *t*, while *α* and *β* are the initial sorption concentration rate (mg·g^−1^·min^−1^) and desorption constant (g/mg), respectively	*α*	1.27 × 10^4^	1.28 × 10^4^
	*β*	0.241	0.201
	*R* ^2^	0.7921	0.8238
Weber–Morris intraparticle diffusion model $q_{t}=K_{I}t^{0.5}+C$, where *K_I_* is the intraparticle diffusion rate constant (mg·g^−1^·min^−0.5^) and *C* is the boundary thickness effect	*K_I_*	2.622	2.769
	*C*	33.44	38.76
	*R* ^2^	0.5877	0.6084

## Data Availability

All the data used in this study are available within this article. Further inquiries can be directed to the authors.

## Sample Availability

Not available.
